# A microfabricated low-profile wideband antenna array for terahertz communications

**DOI:** 10.1038/s41598-017-01276-4

**Published:** 2017-04-28

**Authors:** K. M. Luk, S. F. Zhou, Y. J. Li, F. Wu, K. B. Ng, C. H. Chan, S. W. Pang

**Affiliations:** 1 Department of Electronic Engineering, City University of Hong Kong, Hong Kong, Hong Kong; 2 Center for Biosystems, Neuroscience, and Nanotechnology, City University of Hong Kong, Hong Kong, Hong Kong; 3 The Institute of Lightwave Technology, Beijing Jiaotong University, Beijing, China

## Abstract

While terahertz communications are considered to be the future solutions for the increasing demands on bandwidth, terahertz equivalents of radio frequency front-end components have not been realized. It remains challenging to achieve wideband, low profile antenna arrays with highly directive beams of radiation. Here, based on the complementary antenna approach, a wideband 2 × 2 cavity-backed slot antenna array with a corrugated surface is proposed. The approach is based on a unidirectional antenna with a cardiac radiation pattern and stable frequency characteristics that is achieved by integrating a series-resonant electric dipole with a parallel-resonant magnetic dipole. In this design, the slots work as magnetic dipoles while the corrugated surface radiates as an array of electric dipoles. The proposed antenna is realized at 1 THz operating frequency by stacking multiple metallized layers using the microfabrication technology. S-parameter measurements of this terahertz low-profile metallic antenna array demonstrate high efficiency at terahertz frequencies. Fractional bandwidth and gain are measured to be 26% and 14 dBi which are consistent with the simulated results. The proposed antenna can be used as the building block for larger antenna arrays with more directive beams, paving the way to develop high gain low-profile antennas for future communication needs.

## Introduction

To support the advancement of terahertz (THz) systems for wireless communications^1, 2^, sophisticated antennas are in urgent need. Due to the limited power of commercial THz sources, high gain antennas are required. Although high-performance wideband horn antennas are now available^3^, they are bulky in structure even at very high frequencies. Instead, low-profile antennas with directive beams are demanded for ease of integration with radio-frequency circuits for achieving compact transceiver structures. At microwave frequencies, this can be realized with printed dipole antenna arrays^4^, microstrip antenna arrays^5^, leaky-wave antennas^6^, Fresnel–zone plate antennas^7^, Fabry-Perot antennas^8^, or metasurface antennas^9^, using conventional photolithography techniques. Microfabrication technologies similar to those used in the semiconductor industry have been applied to form on-chip integrated antenna arrays on silicon (Si) substrates^10–12^. However, these integrated chips often suffer from drawbacks such as low efficiency or gain, substrate modes, and back radiation related to the low resistivity and high relative permittivity of Si. At THz frequencies, however, most of these designs are not applicable due to the difficulties encountered in conventional integrated circuit (IC) fabrication. The realization of on-chip planar antenna array remains to be challenging even when advanced IC technologies in Si are used. The high frequency antennas require thick layer of metal to conformally cover substrate material with low permittivity. Therefore special fabrication technologies will be needed to generate microstructures in materials with low permittivity and thickness up to hundreds of micrometers (µm), very different from the thin film (few µm) technologies typically employed in Si-based IC manufacturing. Moreover, development of new technologies will be needed to deposit thick layer of metal to the order of several skin depth uniformly and continuously over hundreds of µm deep microstructures. A number of THz nano-antenna arrays for terahertz spectroscopy have been proposed recently based on slots^13^, printed dipoles^14^ or patch antennas^15^ with simple structures. Some beamforming works that are based on array chips fabricated in Si and operated at lower THz frequencies are also reported^16, 17^.

Patch antennas and arrays have been extensively investigated in the past 4 decades. They have the advantage of low profile in structure, enabling them to be installed conformably to mounting structures or devices. However, they are inherently narrow in bandwidth. Although various bandwidth enhancement techniques have been successfully developed, including the use of stacked patches^18^, a U-slot patch^19^ or an L-shaped probe feed^20^, the radiation characteristics of the antenna vary substantially over the operating bandwidth. The same difficulty is also encountered in designing wideband reflector-backed dipole arrays^21^ or cavity-backed slot arrays^22^.

By integrating a quarter-wave patch antenna with a reflector-backed dipole, a wideband unidirectional antenna element was proposed 10 years ago by Luk and Wong^23^. Such antenna element showed advantages including wide impedance bandwidth, low in cross-polarization, low in backward radiation, and symmetrical radiation patterns. More importantly, the gain and beamwidth of the antenna have little variation over the operating frequency range, which are preferable features for many wireless applications. The antenna, designated as the magneto-electric dipole^24^, is a special kind of complementary antenna consisting of an electric dipole and a magnetic dipole^25^, co-located with orthogonal directions, and with the electric dipole operated in series resonance and the magnetic dipole operated in parallel resonance. When the two dipoles are connected in parallel, the resulting antenna performs with wider bandwidth than that of the original dipoles.

The magneto-electric dipole concept has been successfully applied for developing high performance wideband antenna array for microwave mobile communications and 60 GHz millimeter-wave wireless systems. For THz operation, however, their structures are too complicated to be realized by conventional fabrication techniques.

In this paper, based on the powerful magneto-electric dipole concept and with the objective to develop a simple antenna structure that is easy for microfabrication, an antenna array operated at frequencies around 1 THz is created and demonstrated for the first time. The design, microfabrication technology, and antenna characterization setup are presented. The proposed antenna has many attractive features for future THz communication systems.

## Results

### Design of Single Antenna Element

**Figure 1:**
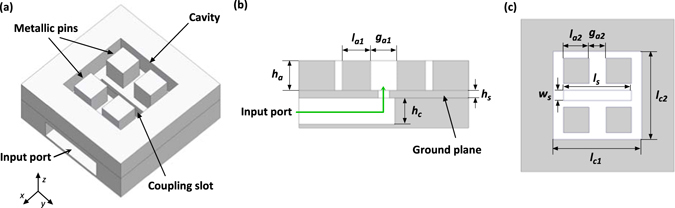
Geometry of the wideband cavity-backed slot antenna with corrugated surface. (**a**) Perspective view, (**b**) side view, and (**c**) top view.

Figure 1a shows the geometry of the proposed antenna element. A horizontal shorted-end air-filled rectangular waveguide is mounted beneath the metallic ground plane for transmitting the input signal. The slot-coupling method which is widely used in antennas working at microwave and millimeter-wave regions^26^ is applied here as the excitation method. The electric signal propagating in the waveguide is coupled to the antenna through a transverse slot etched on the upper surface of the waveguide. The slot-coupling method is promising for THz antenna designs since the simple two-dimensional coupling slot can be realized easily using conventional planar fabrication technologies. There are two pairs of rectangular metallic posts located at the two sides of the coupling slot. These four metallic posts together with the four surrounding sidewalls - resemble a corrugated surface - comprise the radiating aperture of the antenna element. Detailed internal geometries of the antenna are illustrated in Fig. 1b,c. The proposed antenna is simulated using a commercial full-wave electromagnetic simulator, ANSYS high frequency structural simulator (HFSS).

**Figure 2:**
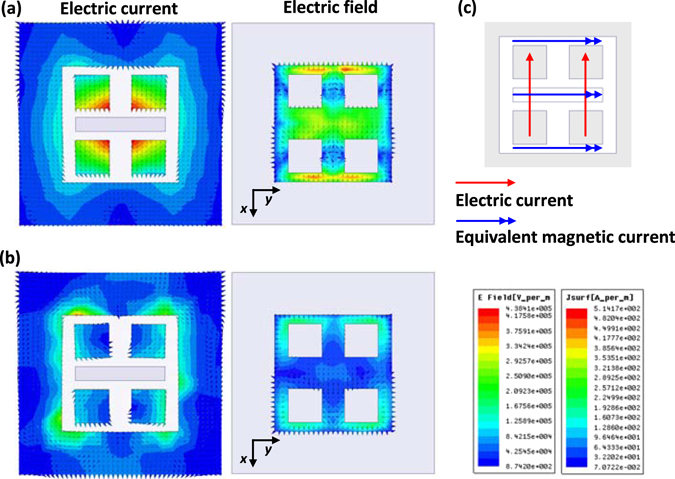
Simulated electric current and field distributions over the radiating aperture of the proposed antenna element. (**a**) The distributions at *t* = 0, (**b**) the distributions at *t* = T/4 where T is the period at 1 THz, and (**c**) radiation mechanism of the antenna element.

In order to understand the operating principle of the proposed antenna, the simulated electric current and field distributions throughout the radiating aperture of the antenna, namely, the top surface of the antenna element are studied. Figure 2a,b show the field distributions at time (*t*) = 0 and T/4, respectively, where T is the period. It can be seen that at *t* = 0, the electric current on the top surface of the four metallic posts is strongly excited with a sinusoidal distribution in the *x*-direction, which is similar to the current distribution on a planar electric dipole. The current on the four sidewalls also contribute to the radiation of the antenna, though its magnitude is much smaller than the counterpart on the metallic posts. In addition, the electric field over the remaining portion of the radiating aperture is also strongly excited. The direction of the electric field is mainly along the *x*-direction, which is the same as that of the electric current. On the other hand, as shown in Fig. 2b, the electric current and field over the radiating aperture at *t* = T/4 are weaker compared with those at *t* = 0. More importantly, the electric current over the left half of the radiating aperture is in opposite direction to the current over the right half. The directions of the electric current on the metallic posts and on the sidewalls are also opposite to each other. Similar results are found for the electric field. As a result, the weak electric current and field with opposite directions over the radiating aperture cannot generate effective radiation at this time.

Based on the above analysis, the working principle of the proposed antenna element can be explained as shown in Fig. 2c. The four top surfaces of the metallic posts work as a pair of electric dipoles along the *x*-direction. The gap between the four metallic posts and the two gaps between the posts and the sidewalls can be regarded as three radiating apertures, which operate as equivalent magnetic dipoles in the*y*-direction in this design. The behavior shown in Fig. 2a,b demonstrate that the electric dipole and the equivalent magnetic dipoles are excited together. According to the study presented in ref. 24, a desirable unidirectional radiation with low backlobe can be realized when the co-located electric and magnetic dipoles, arranged perpendicularly, are fed with the same phase. Therefore, good radiation performance can be fulfilled by properly adjusting the dimensions of the antenna. In this design, in order to achieve effective radiation from the electric dipole above the metallic ground plane, the height of this antenna (*h*_*a*_) is set to be 80 μm, which is approximately a quarter of wavelength at 0.95 THz. It is found in the study that the length of the coupling slot (*l*_*s*_), the width of the gap between the metallic posts (*g*_*a*1_), and the length of the metallic posts (*l*_*a*1_) are the three major parameters that affect the performance of the antenna significantly. A parametric study on the effect of the three parameters on the reflection coefficient of the antenna is shown in Supplementary Fig. 1. The optimized dimensions of the antenna are listed in Supplementary Table 1.

**Figure 3:**
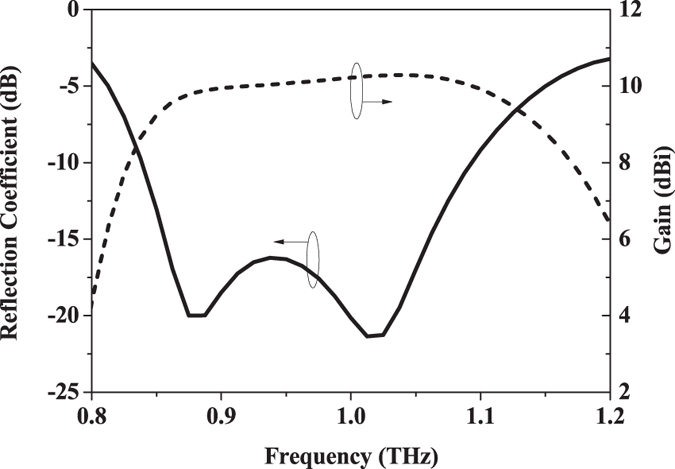
Simulated reflection coefficient and gain of the proposed antenna element.

**Figure 4:**
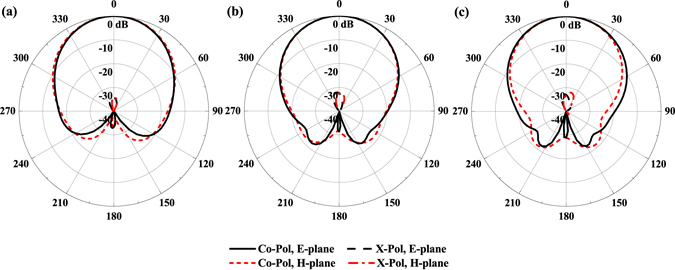
Simulated radiation pattern of the proposed antenna element at different frequencies throughout the operating band. (**a**) *f* = 0.85 THz, (**b**) *f* = 0.95 THz, and (**c**) *f* = 1.05 THz.

**Figure 5:**
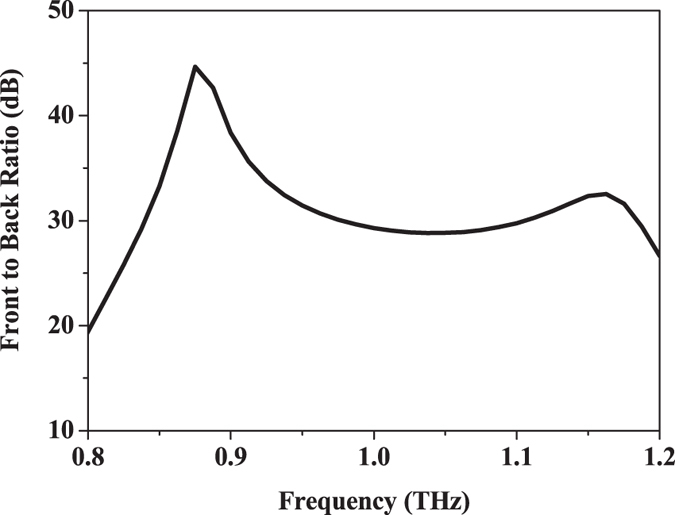
Simulated front-to-back ratio of the proposed antenna element.

Figure 3 shows the simulated reflection coefficient and gain of the proposed antenna element. The −10 dB bandwidth of the antenna is 250 GHz, which is about 26% of the center frequency (standing-wave ratio, SWR <2 from 0.84 to 1.09 THz). There are two resonances over the entire operating frequency band, where the one at lower frequency is mainly determined by the height of the metallic posts and the one at higher frequency is significantly affected by the gap between the metallic posts according to the analysis shown in the Supplementary Fig. 1. Besides, the length of the coupling slot can also be used to tune the two resonances. The gain of the proposed antenna is around 10 dBi throughout the operating band. The simulated radiation pattern of the antenna is depicted in Fig. 4. It shows a cardiac shape with low backward radiation, which is stable over the entire operating band. In the E- and H- planes, the radiation patterns are almost identical to each other. Additionally, the cross polarization level of the antenna is smaller than −32 dB. As presented in Fig. 5, the front-to-back ratio of the antenna is larger than 25 dB over the whole operating band, which demonstrates the superiority of the complementary source approach. A detailed comparison with the other two antenna elements, namely, the slot antenna and the cavity-backed slot antenna as illustrated in Supplementary Figs 2 to 6. The results show the attractive bandwidth and radiation performance of this new design at THz frequencies.

### Design of 2 × 2 antenna array

**Figure 6:**
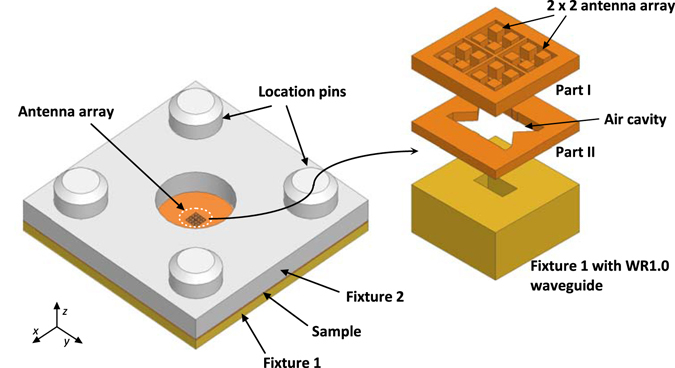
Geometry of the proposed antenna array.

Based on the structure of the antenna element investigated in the previous section, a 2 × 2 array antenna is designed. Figure 6 presents the architecture of the array, which can be divided into two layers, namely, Part I and Part II. Four antenna elements with element spacing of 280 μm comprise the upper layer (Part I). The rectangular metallic posts on the ground plane can be considered as a corrugated surface. In order to excite the four antenna elements with a simple feeding structure, a large air-filled rectangular cavity is mounted below the ground plane of the antenna, which comprises the lower layer of the structure (Part II). A vertical WR1.0 waveguide embedded in the metallic fixture (Fixture 1) is located at the center of the lower surface of the air cavity for antenna excitation. This modified structure allows the antenna to serve as a sub-array for constructing larger arrays excited by a waveguide feed network for possible beamforming. The geometrical dimensions are optimized using the HFSS simulator. Detailed dimensions of the cavity are given in Supplementary Fig. 7 and Supplementary Table 2. Four location pins with a diameter of 2 mm are arranged around the antenna for alignment. An additional metallic fixture (Fixture 2) with a thickness of 1 mm is added above the antenna array in order to guarantee tight connection between the sample and Fixture 1. A hole with a diameter of 3 mm is drilled at the center of Fixture 2 for antenna radiation.

**Figure 7:**
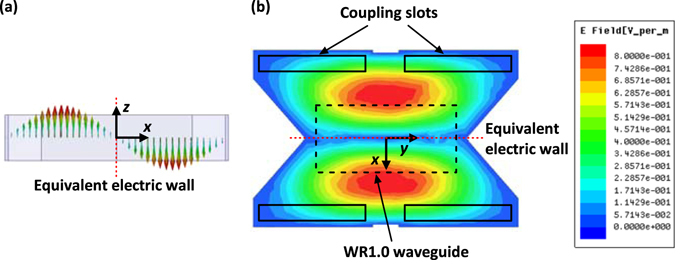
Simulated field distributions in the air cavity. (**a**) Vector distribution and (**b**) magnitude distribution.

The simulated electric field distribution in the air-filled cavity is illustrated in Fig. 7. It can be seen from Fig. 7a that the central-plane of the cavity along the *y*-direction is equivalent to an electric wall. Hence, the direction of the electric field in the upper half of the cavity is opposite to that in the lower half, which results in the required in-phase excitation to the four coupling slots. Besides, as shown in Fig. 7b the magnitude of the field in the cavity is symmetrical in both *x* and *y* directions, hence the four slots can be excited with the same amplitude.

### Microfabrication Technology for THz Antenna

Microfabrication technology was used to generate antenna structures using optical lithography with negative photoresist^27^. For the proposed antenna, it consists of three layers of patterned areas as shown in the perspective and side views in Fig. 6. These three layers were fabricated in two pieces. The first piece (Part I) of the antenna includes two layers where the top layer has patterned areas larger than the bottom layer. The second piece (Part II) of the antenna has patterned areas larger than the bottom layer of the first piece and needs to be fabricated separately.

**Figure 8:**
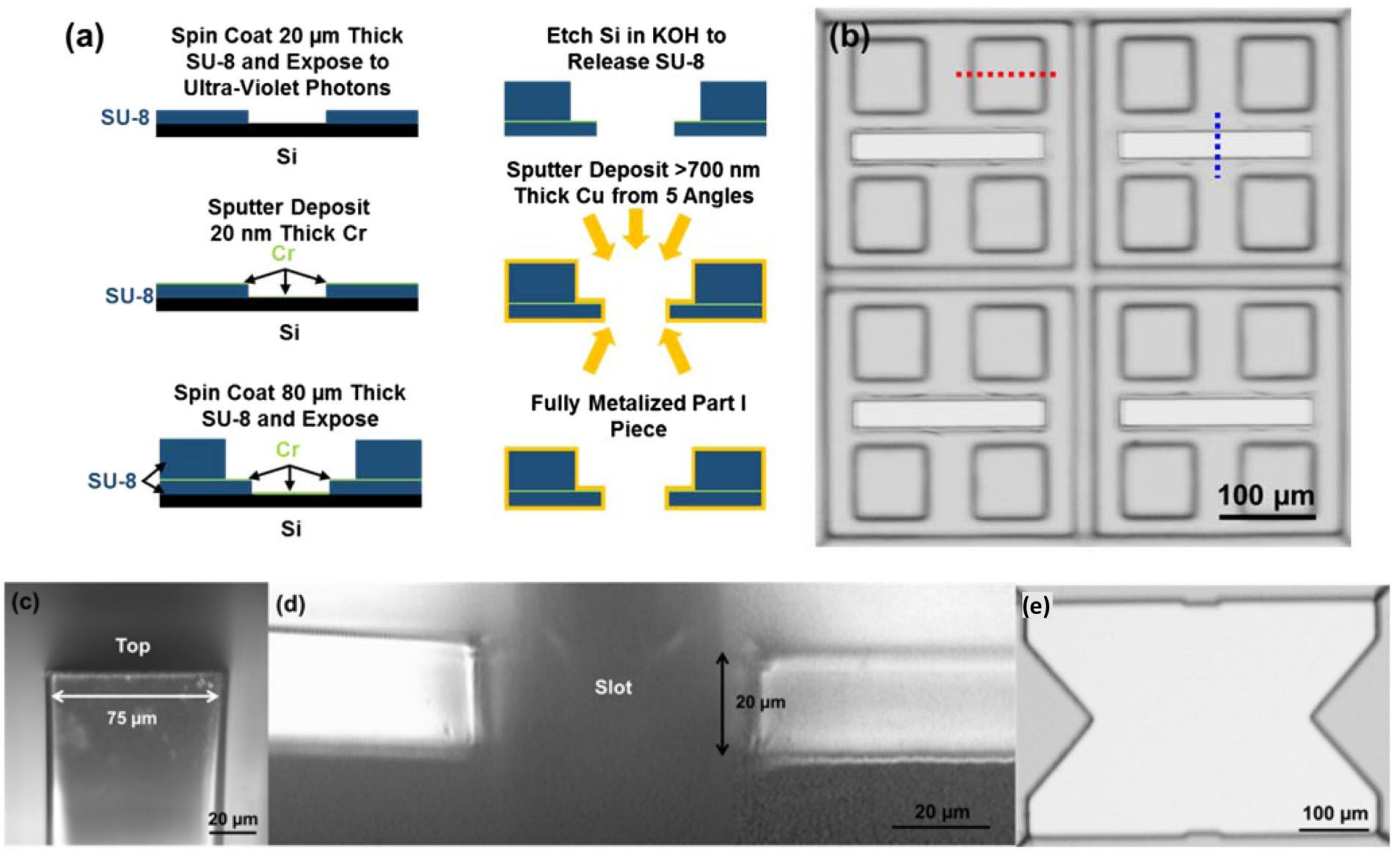
(**a**) Fabrication technology used for double layer structure in the Part I piece. (**b**) Optical micrograph of the Part I piece of the THz antenna. Cross-sections of (**c**) top part of square strut (labelled by red dotted line in (**b**) and (**d**) two sides of slot (labeled by blue dotted line in (**b**). (**e**) Optical micrograph of the Part II piece of the THz antenna.

Figure 8a shows the microfabrication technology used to generate the top and bottom layers (Part I piece) of the antenna. 20 µm thick SU-8 2010 negative photoresist was spin-coated and patterned as the bottom layer on a Si substrate, followed by deposition of a 20 nm thick chromium (Cr) on top. This Cr layer enhanced the visibility of the bottom patterned layer, and made the alignment of the top layer more accurate. Next, an 80 µm thick SU-8 2050 was spin-coated and patterned on top of the Cr layer. The double-layer SU-8 structure with a total thickness of 100 µm was separated by etching the Si substrate with 30% potassium hydroxide (KOH) solution at 25 °C. Finally, the free standing SU-8 double-layer was metallized by sputter-depositing 700 nm thick copper (Cu) to provide >10× the thickness of the skin depth (65.2 nm) at 1 THz. Figure 8b shows the optical micrograph of the fabricated Part I piece. To provide conformal metal coating on all surfaces of this Part I piece, including the top, sidewalls, and bottom of the 100 µm thick double layers, sputter-deposition was carried out from five different angles. The devices were placed at 15° relative to the Cu sputter target. The angle deposition of Cu was performed 4 times to cover all of the 100 µm thick sidewalls of the corrugated surface. The antenna patterns were aligned diagonally to the Cu target and the angle deposition was performed with devices facing up and down, as well as at opposite directions relative to the Cu target. An additional normal incident deposition was performed in order to cover the areas shadowed by the 16 square struts during the angle deposition at 4 different orientations. Figure 8c,d show the cross sections of the top part of a 100 µm thick square strut and two sides of the 40 µm wide slot (labeled by red and blue dotted lines in Fig. 8b, respectively). The results demonstrated the conformal Cu coverage on the top surface and both sidewalls.

The Part II piece was fabricated in a similar way as the Part I piece, except that only a single layer of 70 µm thick SU-8 2050 was spin-coated and patterned. Optical micrograph showing the top view of the fabricated Part II piece is shown in Fig. 8e. For radio-frequency and microwave components, the increased surface roughness could increase the insertion loss when the root mean square (rms) value of the surface roughness is on the same order of skin depth. The rms roughness of the deposited Cu surface was measured by an atomic force microscope (AFM) to be 6.69 ± 0.8 nm, which is approximately one-tenth of the skin depth at 1 THz. Hence the additional insertion loss due to surface roughness of the patterned pieces with sputter-deposited Cu is negligible. Therefore, sputter-deposited Cu has the advantages of having conformal coverage and smooth morphology. In comparison, similar structure metallized by electroplated Cu (Supplementary Fig. 8) had rms surface roughness of 70.7 ± 8.2 nm, which is comparable to the skin depth and could introduce much larger insertion loss.

### Experimental Results

**Figure 9:**
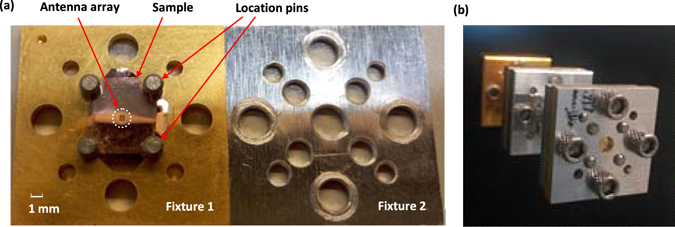
Geometry of the fabricated prototype for measurement. (**a**) Photographs of the fabricated prototype and (**b**) prototype under test.

The fabricated prototype of the THz array with fixtures is illustrated in Fig. 9a. For the measurement, the backside of Fixture 2 is connected to the extended WR 1.0 waveguide flange of a Virginia Diodes Inc. (VDI) extender as shown in Fig. 9b. An Agilent Network Analyzer N8245A with VDI WR1.0 VNA extenders was utilized to measure the reflection coefficient of the antenna. The radiation performance was performed by using an in-house far-field THz antenna measurement system. The gain of the array was obtained by comparing with a standard VDI WR1.0 diagonal horn.

**Figure 10:**
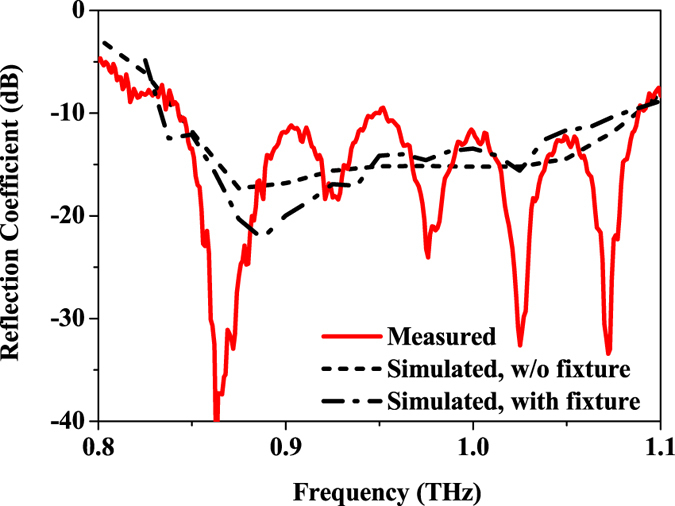
Measured and simulated reflection coefficient of the antenna array.

Measured and simulated reflection coefficients are shown in Fig. 10, which are in good agreement. The measured and simulated −10 dB bandwidths of the antenna array are 26% (from 0.84 to 1.09 THz) and 25% (from 0.84 to 1.08 THz), respectively. It is found that with the existence of Fixtures 2, more resonances are observed on the curve of the reflection coefficient, which is mainly due to the influence of reflection from the possible air gaps around the antenna design drilled in Fixture 2.

**Figure 11:**
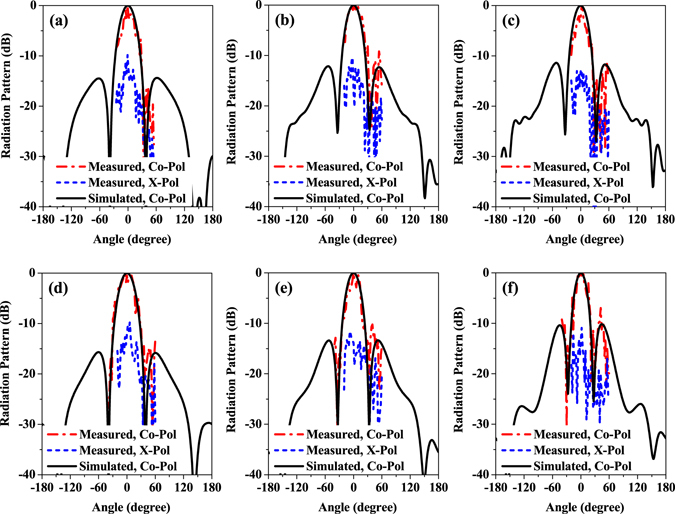
Measured and simulated radiation pattern of the antenna array. (**a**) *f* = 0.85 THz, E-plane, (**b**) *f* = 0.95 THz, E-plane, (**c**) *f* = 1.05 THz, E-plane, (**d**) *f* = 0.85 THz, H-plane, (**e**) *f* = 0.95 THz, H-plane, and (**f**) *f* = 1.05 THz, H-plane.

Figure 11 presents the measured and simulated radiation patterns of the designed antenna array at different frequencies. The measured results agree well with the simulations, which confirm the excellent performance of the proposed antenna structure. The radiation pattern is stable and symmetrical within the operating band of the array. Due to the unpredictable reflection from the fixtures and measurement setup near the antenna under test, a series of small ripples can be seen in the measured results. In addition, the undesirable reflection also increases the cross polarization level in the measurement. The measured cross polarization is less than −10 dB, which is larger than the simulated one.

**Figure 12:**
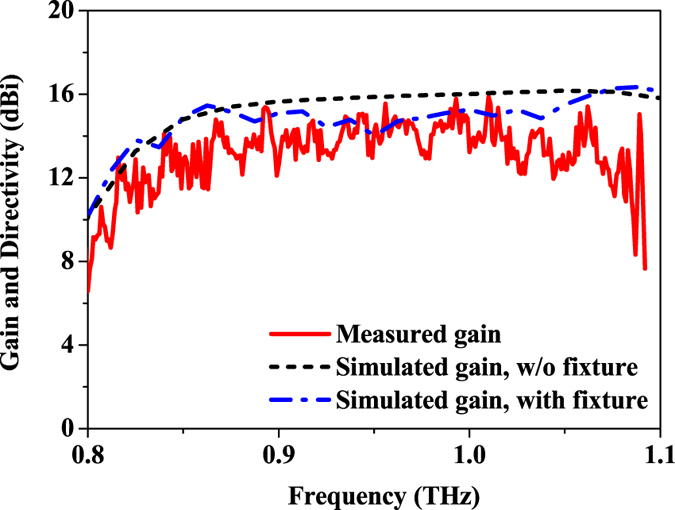
Measured and simulated gain of the antenna array.

The gain of the antenna array at broadside direction is illustrated in Fig. 12. Simulated gain of up to 16 dBi with a variation of less than 1.8 dB is achieved by the design without fixtures (the configuration shown in Fig. 6). With the existence of the fixtures, the gain decreases to around 14 dBi over the operating band. By comparing the radiation patterns of the antennas with and without the fixtures as shown in Supplementary Fig. 9, it is found that the reflection from the fixtures could influence the shape of the radiation pattern. The sidelobe level is increased and the maximum of the radiation pattern may also move away from the broadside direction. Therefore, the change in the radiation pattern may lead to the decrease in the antenna gain. Moreover, the measured gain of the design is slightly lower than the simulated one with fixtures, which is mainly caused by the unpredictable metallic loss of the fabricated sample and the fixtures.

**Figure 13:**
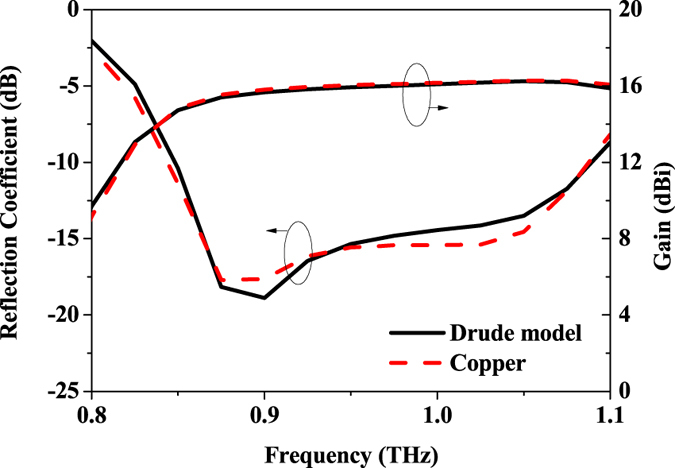
Comparison of simulated results for the antenna array using the ideal copper model and the Drude model.

Usually, ideal metallic materials are assumed in the simulation of antennas in the microwave and millimeter-wave frequency ranges. At higher frequencies, plasmons can be excited at the surface of a metallic material, especially when the operating frequency approaches the optical regimes, which means that the ideal metallic model is not applicable any more. In order to investigate the possible plasmonic phenomenon occurred at THz frequencies, the Drude model is applied to characterize the property of the copper material. A comparison of the simulated results for the reflection coefficient and gain obtained from the two models is illustrated in Fig. 13. Very small discrepancy can be seen between of the two models, which demonstrates that there is insignificant effect of surface plasmons for this antenna design operated at frequencies up to 1-THz.

The measured angle is limited to ±60° due to the limited length of the coaxial cables that are connected to the VDI modules. There are 2 pieces of radio frequency (RF) cables carrying the RF and local oscillator (LO) signals to each VDI module for frequency up/down-conversion up to 1.0 THz. The two cables are very sensitive to bending or stretching. These sets of cables have to operate within their designated blending radius to provide stable phase and amplitude performance during measurement. Once the VDI module (the right one, Rx, in Supplementary Fig. 11) reaches over ±60°, the cables are over-stretched which would affect the measured signal phase and amplitude. Therefore, the optimized measured angular range for this 1.0 THz far-field platform is 120° in total.

The calibration is performed by VDI WR 1.0 Calibration kit. We perform the full two ports short-open-through (SOT) calibration method. Each of the 1.0 THz VDI modules is pre-installed with a straight waveguide (WR1.0SWG1R4) as the interface to the antenna under test (AUT) for radiation pattern measurement as well as the standard gain horn (SGH) for gain comparison. We include both VDI-waveguide feeds during calibration. Therefore, the calibrated reference planes during radiation pattern and gain measurements are exactly the same as the calibration. No de-embedding is required here. In addition, we covered all the metallic surfaces with absorbers during measurement in order to minimize any multiple-reflection.

## Discussion

Horn antennas are available commercially for terahertz applications, which work with a single mode for ease of fabrication. One of the disadvantages of these horn antennas is the high sidelobe radiation due to the uniform aperture field distribution.

In order to meet the requirements of various applications, an antenna array is a powerful approach to realize low-profile wideband microwave and millimeter-wave antennas with high directive radiation, adjustable beam shape, and side lobe levels. However, it was not employed to develop THz antennas due to the difficulty to fabricate complex antenna structure at sub-micrometer level. It was also believed that metallic antenna arrays operated at terahertz frequencies were low in efficiency because of the metallic losses, which increase with frequencies.

A 2 × 2 antenna array operated at 1 THz has been designed, fabricated and tested for the first time. The antenna array exhibits more than 250 GHz bandwidth and 14 dBi peak gain. This result demonstrates that a metallic antenna array can be realized at such high frequencies with high antenna efficiency and wide bandwidth if appropriate antenna structure is designed and small roughness of the metallic surfaces can be achieved.

In order to fully utilize the available wide bandwidth of terahertz frequencies, the magneto-electric dipole antenna concept was employed. For ease of realization using microfabrication techniques, the antenna array developed here has a simple structure that is similar to the conventional cavity-backed slot array except that the planar surface for the slots is replaced by a corrugated surface. Such a replacement results in the increase in the antenna bandwidth and the improvement in the antenna radiation pattern. As the antenna array is center-fed by a waveguide opening, it can be used as a sub-array for constructing larger arrays, as shown in Supplementary Fig. 10. The waveguide feed network originally designed in lower frequency bands, such as the double-layered full-corporate feed network^28, 29^, can be utilized to excite a series of sub-arrays investigated in this paper simultaneously for various applications. The antennas can also be used for terahertz sensing and imaging, in addition to wireless communications.

Since the entire outer surface of the double layer SU-8 structure of the proposed antenna element is metallized by depositing a thick layer of copper film to the order of several skin depth, the dielectric loss is minimized in this design. In addition, the smooth metallic surface with a roughness of much less than the skin depth at 1 THz lowers the possible metallic loss for this design. As a result, promising radiation characteristics can be achieved in this study.

## Method

### Full-Wave Electromagnetic Simulation

Commercial full-wave electromagnetic simulator ANSYS HFSS was applied to study the performance of the proposed antenna element and array. The simulation model was generated in the simulation software according to the configurations given in Figs 1 and 6 with copper as the antenna material.

In order to investigate the possible plasmon phenomenon within the operating frequency band of the design, simulation model based on the Drude model was also generated. The dielectric constant of the metallic material used in the model can be expressed as

$${\rm{\varepsilon }}(\omega )=1-\frac{{\omega }_{p}^{2}}{{\omega }^{2}+i\gamma \omega }$$

where ${\omega }_{p}$ is the plasma frequency of a metallic material, *ω* is the angular frequency, and $\gamma$ is the damping constant. The values of these parameters for copper can be found in ref. 30.

### Antenna Characterization

The input impedance of the fabricated antenna was measured by an Agilent Vector Network Analyzer N5245A connected to a WR01.0 VDI network analyzer extension modules covering the frequency band from 0.75 to 1.1 THz. The radiation patterns of the prototype were measured by an in-house far-field terahertz band antenna measurement system as depicted in Supplementary Fig. 11.

The platform is composed of three major parts, namely, (i) a pair of WR 01.0 VDI network analyzer extenders as a THz signal source for transmission test (S21), (ii) a manual mechanical rotational stage, and (iii) a laser alignment system. The whole platform is installed on a Newport “M-SG-45-4” honeycomb optical breadboard (1500 mm × 1200 mm × 110 mm) for providing vibration-free testing environment as shown in Supplementary Fig. 12. The antenna under test (AUT) as the transmitting antenna with fixtures is mounted on the VDI module pointing toward the center of the rotation. The rotational stage is manually operated which can rotate in angular direction up to ±90° horizontally with a precision of 1°. All the horizontal and vertical alignments are achieved by using a 2-axis laser tracker.

A standard horn antenna with a nominal antenna gain of 22 dBi at the frequency range of 0.75–1.1 THz is used for the receive antenna at a far-field distance of 159 mm (159λo in separation at 1.0 THz). The receive horn antenna is connected to the WR01.0 VDI network analyzer extension module for receiving the RF power from the AUT. RF absorbers are used around the platform. For the gain measurement, two identical standard gain horns from VDI, WR 01.0 Diagonal Horn, with frequency range of 0.75 to 1.1 THz, are employed for making the direct gain comparison to retrieve the gain value of the proposed antenna.

## Electronic supplementary material

Supplementary Information
