# Supplementary material for: A microfabricated low-profile wideband antenna array for terahertz communications
Source: Sci Rep. 2017 Apr 28;7:1268. doi: 10.1038/s41598-017-01276-4 (PMC5430892; doi:10.1038/s41598-017-01276-4)

# A microfabricated low-profile wideband antenna array for terahertz communications

K. M. Luk[1], S. F. Zhou[1,2]*, Y. J. Li[1,3]*, F. Wu[1], K. B. Ng[1], C. H. Chan[1], and S. W. Pang[1,2]*

[1] Department of Electronic Engineering, City University of Hong Kong, Hong Kong

[2] Center for Biosystems, Neuroscience, and Nanotechnology, City University of Hong Kong, Hong Kong

[3] Current Address: The Institute of Lightwave Technology, Beijing Jiaotong University, Beijing, China

# Supplementary Materials

## A. Parametric studies of the single antenna element

Parametric studies of the length of the coupling slot $l_s$, the width of the gap between the metallic posts $g_{a1}$, and the length of the metallic posts $l_{a1}$ are given in Supplementary Fig. 1. There are two resonances occurred to produce the operating band of the proposed antenna. It is seen that $l_s$ can influence both the higher and the lower resonances significantly. On the other hand, the higher resonance can also be controlled by $g_{a1}$, while the lower one is more sensitive to $l_{a1}$. Therefore, by fine tuning the values of the three parameters, good impedance matching of the proposed antenna element can be achieved. The final dimensions of the fabricated antenna are list in Supplementary Table 1.

**Supplementary Figure 1. Simulated reflection coefficient of the single antenna element with different** (a) $l_s$, (b) $g_{a1}$, and (c) $l_{a1}$.

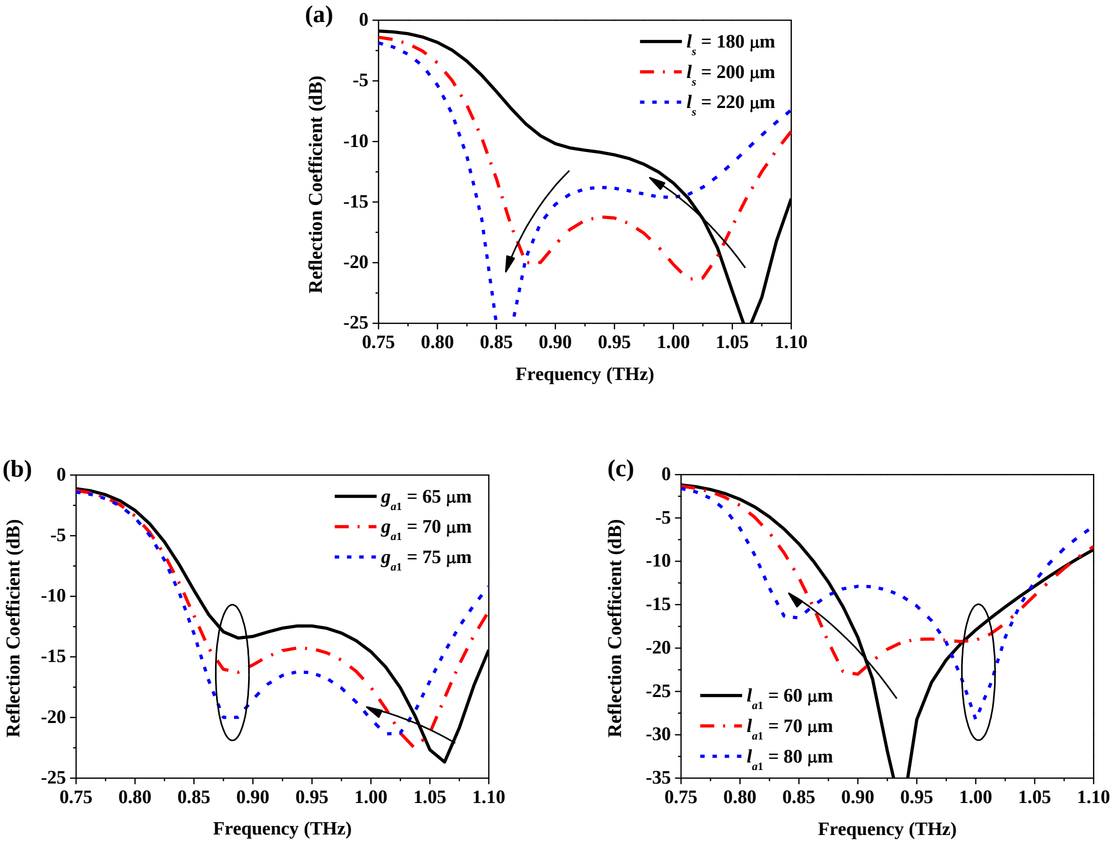

**Supplementary Table 1. Values of Dimensions of the THz Antenna (Units: μm)**

| Parameter | $h_a$ | $h_s$ | $h_c$ | $l_{a1}$ | $l_{a2}$ | $g_{a1}$ |
|---|---|---|---|---|---|---|
| Value | 80 | 20 | 70 | 75 | 75 | 70 |
| Parameter | $g_{a2}$ | $l_{c1}$ | $l_{c2}$ | $l_s$ | $w_s$ | |
| Value | 50 | 260 | 260 | 200 | 30 | |

## B. Comparison of performance of three kinds of antenna elements

Three types of antenna elements are illustrated in Supplementary Fig. 2, where the third one (Supplementary Fig. 2c) is the proposed antenna, while the other two (Supplementary Fig. 2 a,b) are used for comparison. As shown in Supplementary Fig. 3, only one resonance can be seen for the slot antenna and the cavity-backed slot antenna, resulting in much narrower impedance bandwidths as compared to the proposed design. The simulated radiation patterns of the three kinds of antennas at the center operating frequency are exhibited in Supplementary Fig. 4. It can be seen that the radiation patterns of the proposed design are almost identical in the two orthogonal planes, which is desirable for the antenna arrays. Besides, better front-to-back ratio can be achieved by the proposed antenna as given in Supplementary Fig. 5. The simulated gain of the slot antenna is about 4 dB lower than the other two kinds of antenna elements as shown in Supplementary Fig. 6. Although the maximum gain of the cavity-backed slot antenna is similar to the proposed antenna, the latter one has the advantage of stable gain behavior over a wide bandwidth.

**Supplementary Figure 2. Geometries of the three kinds of antenna elements.** (a) The slot antenna, (b) the cavity-backed slot antenna, and (c) the proposed antenna.

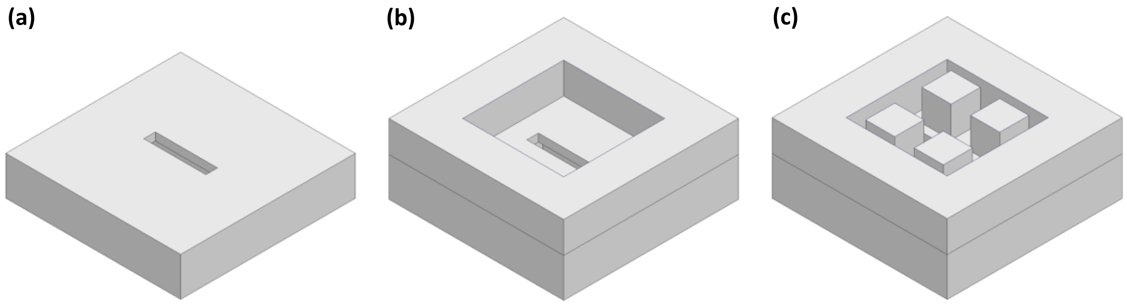

**Supplementary Figure 3. Simulated reflection coefficients of the three kinds of antenna elements.**

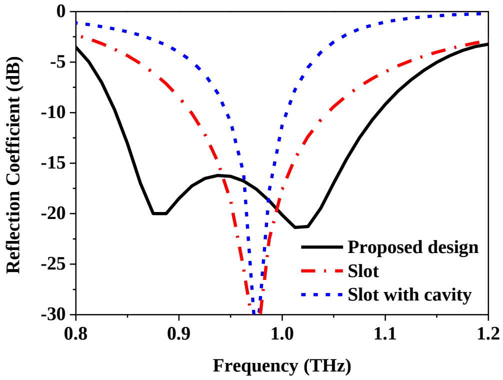


**Supplementary Figure 4. Simulated radiation patterns of the three kinds of antenna elements.** (a) The slot antenna, (b) the cavity-backed slot antenna, and (c) the proposed antenna.

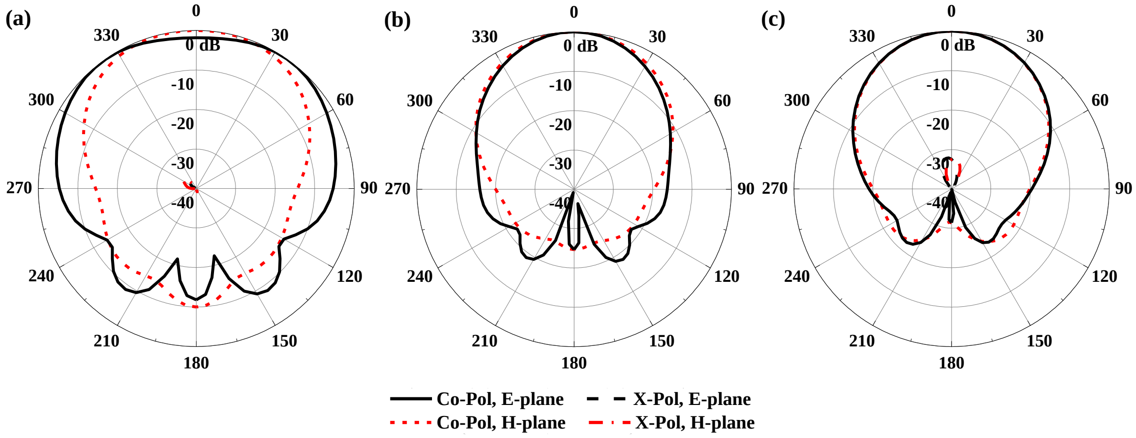

**Supplementary Figure 5. Simulated front-to-back ratios of the three kinds of antenna elements.**

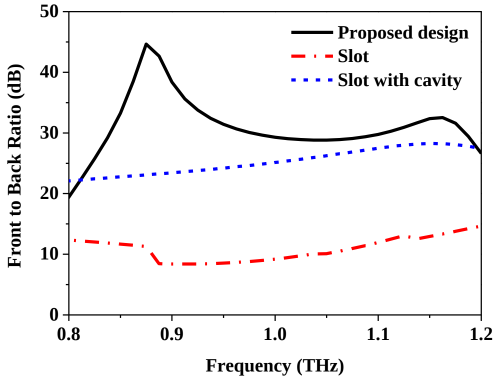


**Supplementary Figure 6. Simulated gains of the three kinds of antenna elements.**

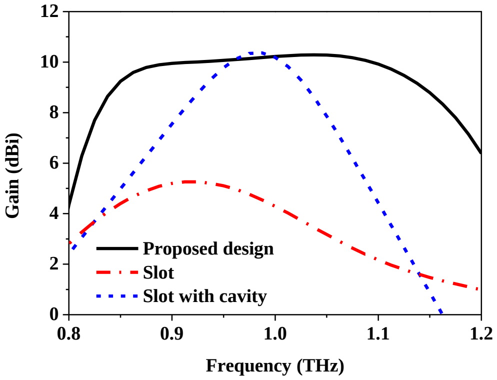


## C. Dimensions of the air cavity of the antenna array

The configuration of the air-filled cavity as the feeding structure of the antenna array is illustrated in Supplementary Fig. 7 along with detailed dimensions. The final values of the dimensions are listed in Supplementary Table 2.

**Supplementary Figure 7. Geometry of the air cavity with detailed dimensions.**

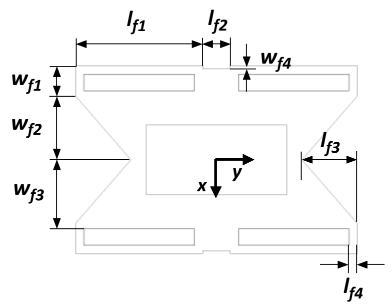


**Supplementary Table 2. Values of Dimensions of the Air Cavity (Units: µm)**

| Parameter | $l_{f1}$ | $l_{f2}$ | $l_{f3}$ | $l_{f4}$ | $w_{f1}$ | $w_{f2}$ | $w_{f3}$ | $w_{f4}$ |
|---|---|---|---|---|---|---|---|---|
| Value | 230 | 50 | 100 | 15 | 55 | 115 | 125 | 5 |

## D. Fabricated prototype of a corrugated surface structure

Supplementary Fig. 8 gives the optical micrograph of the corrugated surface structure metallized by electroplated Cu by microfabrication technology.

**Supplementary Figure 8. 100 µm thick freestanding microstructures in SU-8 covered by 3 µm thick electroplated Cu.**

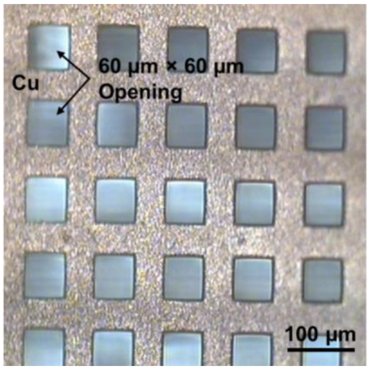

## E. Simulated radiation patterns of the antenna arrays with and without fixtures

**Supplementary Figure 9. Comparison of the simulated radiation patterns of the antenna arrays with and without fixtures.** (a) $f$ = 0.85 THz, E-plane, (b) $f$ = 0.95 THz, E-plane, (c) $f$ = 1.05 THz, E-plane, (d) $f$ = 0.85 THz, H-plane, (e) $f$ = 0.95 THz, H-plane, and (f) $f$ = 1.05 THz, H-plane.

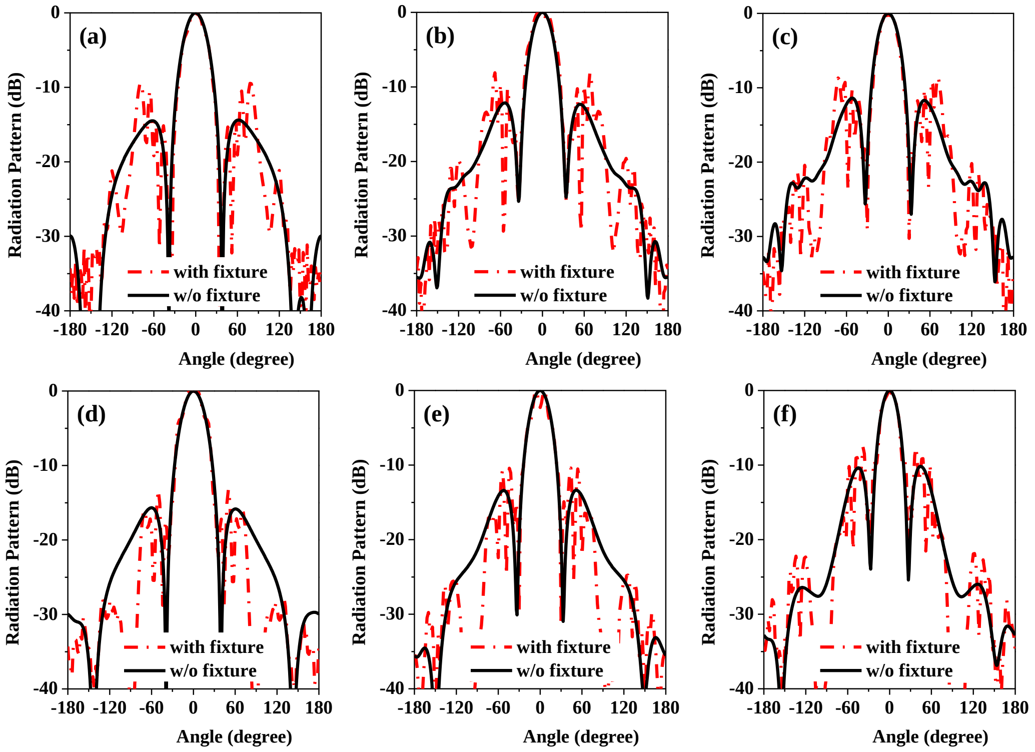


## F. Layout of a larger array composed of the proposed antennas as sub-arrays

**Supplementary Figure 10. Schematic of a 4 × 4 ME-dipole antenna array with waveguide feeds.**

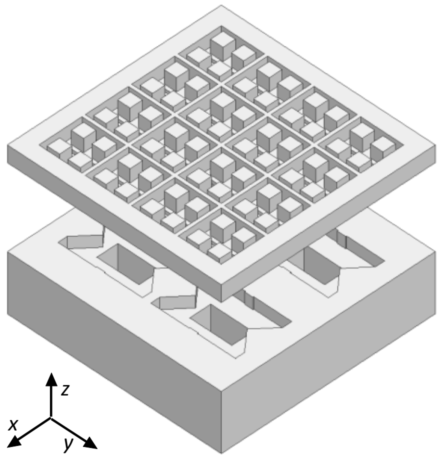

## G. Antenna measurement systems

**Supplementary Figure 11. In-house far-field terahertz band antenna measurement system.**

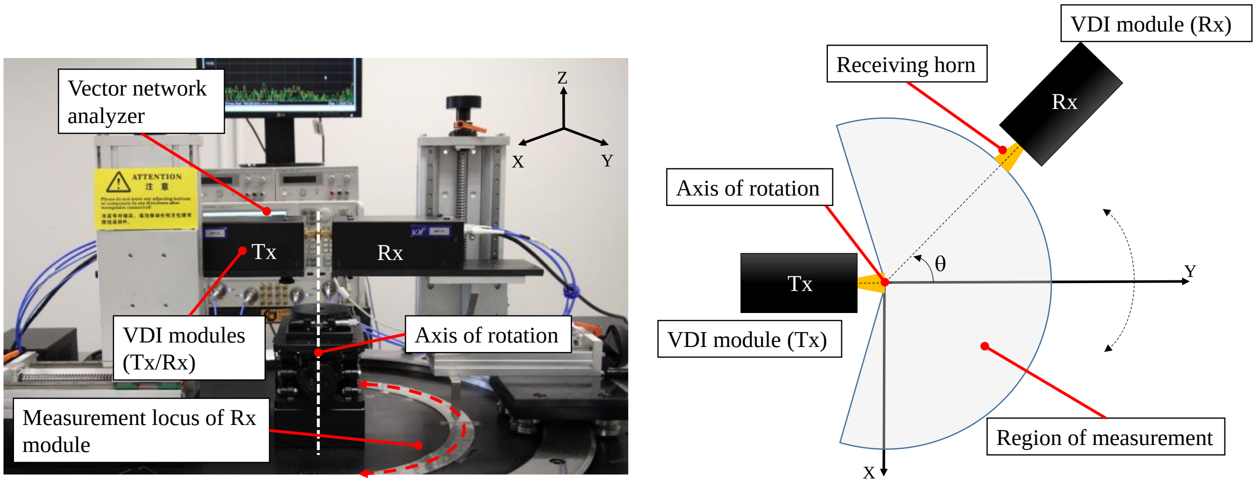


**Supplementary Figure 12. Proposed antenna with mounting fixture under testing.**

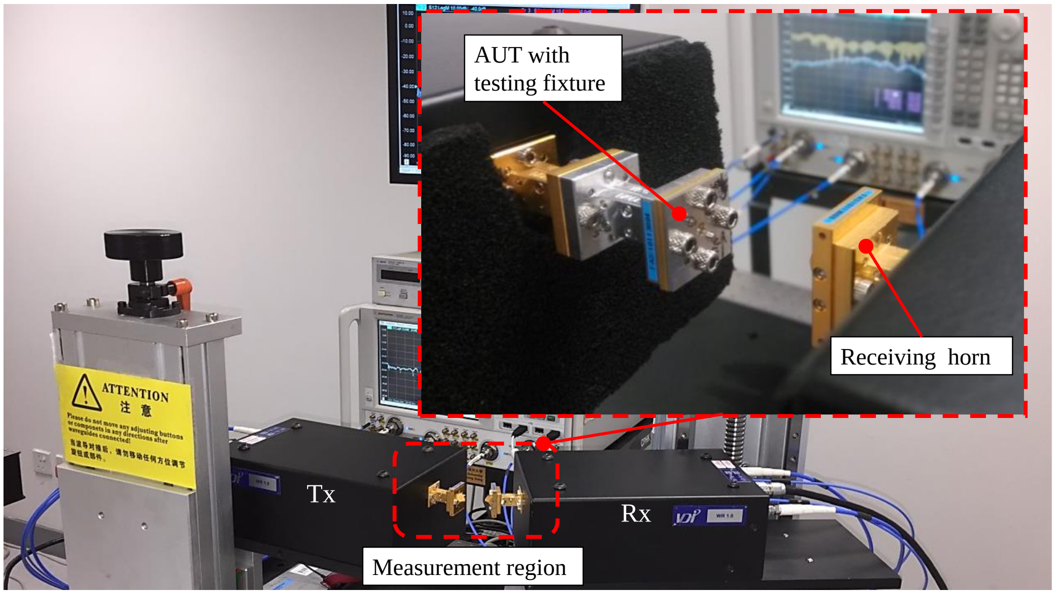

Supplement: Supplementary file 1 — Supplementary Information [file 41598_2017_1276_MOESM1_ESM.pdf]
